# Safety Framework for Gastric Mucosal Ablation (GMA) Using Hybrid Argon Plasma Coagulation (hAPC): International Expert Consensus

**DOI:** 10.1007/s11695-026-08685-3

**Published:** 2026-05-16

**Authors:** Vivek Kumbhari, Adrian Sartoretto, Ivo Boskoski, Diogo de Moura, Dilhana Badurdeen, Daniel Maselli, Eduardo de Moura, Valerio Pontecorvi, Barham Abu Dayyeh, Christopher McGowan

**Affiliations:** 1 Division of Gastroenterology and Hepatology, Mayo Clinic, Florida, USA; 2 The BMI Clinic, Double Bay, NSW, Australia; 3 Digestive Endoscopy Unit, Fondazione Policlinico Universitario Agostino Gemelli IRCCS, Rome, Italy; 4 Department of Gastroenterology, Hospital das Clínicas da Faculdade de Medicina da Universidade de São Paulo, São Paulo, Brazil; 5 Instituto DO`R de Ensino e Pesquisa, São Paulo, Brazil; 6 Department of Gastroenterology, Mayo Clinic, Rochester, USA; 7 D’Or Institute for Research and Education, Rio de Janeiro, Brazil; 8 True You Weight Loss, Cary, NC USA

**Keywords:** Argon plasma coagulation, Gastric mucosal ablation, Obesity, Endoscopic metabolic therapy, Hybrid APC, Procedural safety

## Abstract

**Background:**

Gastric mucosal ablation (GMA) using hybrid argon plasma coagulation (hAPC) is an investigational endoscopic technique that selectively devitalizes ghrelin-producing gastric mucosa to modulate appetite-regulating pathways. As clinical investigation extends beyond early trials, a standardized safety framework is needed to support responsible implementation.

**Methods:**

An international panel of investigators with direct procedural experience in GMA (> 300 procedures collectively) developed consensus safety recommendations using a modified Delphi process. Formal systematic review with GRADE assessment was not undertaken, as the nascent evidence does not support such evaluation. Consensus was defined as ≥ 75% agreement among panelists. Recommendations were classified by consensus strength (high ≥ 80%, moderate 60–79%, low 51–59%) reflecting the degree of panel agreement rather than formal certainty of evidence.

**Results:**

The panel identified nine procedural and post-procedural domains important for the safe execution of GMA. These include: (1) cold saline submucosal injection to elevate the mucosa; (2) ablation precisely confined to the elevated zone; (3) ablation limited to 5 mm from the edge of each fluid cushion; (4) APC energy delivery limited to ≤ 40 W; (5) judicious evacuation of argon gas throughout the procedure; (6) high-dose proton pump inhibitor therapy for a minimum of four weeks; (7) sucralfate for four weeks; (8) gradual post-procedure dietary advancement; and (9) operator credentialing, including proctoring until proficiency is demonstrated.

**Conclusions:**

This consensus-driven framework for GMA provides preliminary safety guidance for clinical investigation and may serve as a model for other emerging endoscopic metabolic therapies.

**Supplementary Information:**

The online version contains supplementary material available at 10.1007/s11695-026-08685-3.

## Key Points

Nine safety domains provide preliminary safety guidance for gastric mucosal ablation.

Submucosal injection and ≤ 40 W APC power are mandatory safety measures.

Serious adverse event rate was 1.1% (4/374 procedures) in pooled analysis.

Risk mitigation bundle implementation eliminated Grade III+ events.

**Supplementary Information:**

The online version contains supplementary material available at 10.1007/s11695-026-08685-3.

## Introduction

 Gastric mucosal ablation (GMA; previously termed gastric mucosal devitalization, GMD) is an emerging and investigational endoscopic technique aimed at devitalizing hormonally active mucosa on the greater curvature of the gastric body and the gastric fundus (when ablation is limited to the fundus, the term fundal GMA [fGMA] is used), replacing it with fibrosed mucosa. Ablation of ghrelin-producing cells reduces appetite and promotes weight loss [1, 2]. GMA offers a minimally invasive, organ-sparing alternative to metabolic surgery, aiming to remodel the stomach to functionally and anatomically resemble a surgical sleeve gastrectomy. Its therapeutic rationale is supported by preclinical and clinical data showing that selective mucosal devitalization reduces body weight, suppresses circulating ghrelin, improves metabolic indices, and modulates vagal and hormonal pathways involved in appetite regulation [1–6].

GMA remains an investigational procedure. The current clinical evidence is derived primarily from small, single-arm studies and pilot registries, with follow-up limited to 6–12 months. Randomized controlled trials comparing GMA with sham or standard care have not yet been completed. This consensus focuses on procedural safety parameters to support standardized, responsible clinical use and continued evidence generation, without pre-empting future determinations regarding broader clinical adoption.

The technique is most commonly performed using hybrid argon plasma technology (HybridAPC^®^ or MOVIVA^®^, Germany), which combines submucosal saline injection with argon plasma coagulation (APC). This approach enables wide-field mucosal ablation while protecting deeper layers of the gastric wall, thereby mitigating the risk of thermal injury to the muscularis propria [3]. Initial results suggest that GMA may have potential both as a standalone or combination procedure with endoscopic sleeve gastroplasty (ESG), and as a primary or secondary weight-loss treatment modality [3, 4, 6–8]. 

As clinical investigation of GMA expands beyond initial pilot studies, standardized procedural safeguards are important to mitigate the risk of complications such as deep tissue injury, ulceration, delayed hemorrhage, and perforation [9, 10]. In response, we have convened an international panel to develop a structured safety framework. This report presents the resulting expert consensus, integrating preclinical and clinical insights into actionable recommendations designed to optimize the safety profile for this promising endoscopic metabolic and bariatric therapy. This framework is intended to guide safety practices within clinical investigation and should not be interpreted as endorsing the routine clinical adoption of GMA outside of structured research settings.

**Table:** 

9 Tenets for Safe GMA
1. **Lift before you fire**- Inject cold (6–8 °C) saline submucosally before every APC application to raise the mucosa and insulate deeper wall layers.
2.** Only treat what lifts** - Creat a clear submucosal cushion. If it won't elevate , skip that area. Note: scar tissue transmits heat unpredictably.
3.** Leave a safety rim**- Ablate up to 5mm from the cushion edge to protect the transition zone. Reinject adjacent areas and ablate to coalesce and achieve a uniform treatment field.
4.** Low power is enough** – Set to Pulsed APC ≤ 40 W. In the thin walled gastric fundus, in particular, 30-35 W is sufficient (white/brown color of the mucosa).
5. **Mind the gas** - Frequent suctioning of intragastric argon is important to prevent overdistention and gas-related complications. Use a dual-channel endoscope and/or endoscopic pressure regulator if available.
6. **Block the acid** – Prescribe a high-dose PPI (e.g., omeprazole 40 mg BID) for a 8-12 weeks post-procedure.
7. **Add a barrier** – Give sucralfate 1 g four times daily for 4 weeks to coat and protect the ablated surface.
8.** Advance the diet gradually** – 72 h clear liquids → full liquids → puréed foods → soft solids.
9. **Get trained** – Perform at least 3–5 proctored HybridAPC GMA cases before working independently.

## Consensus Development Methods

This expert guidance was developed by an international panel composed of gastroenterologists who have participated in one or more clinical studies evaluating GMA with hAPC. Panelists represented investigational centers across North America, Europe, Australia, and South America.

### Evidence Review

All available clinical, preclinical, and translational data on GMA were reviewed. This included ten prospective studies (both completed and ongoing: see Table 1) [3, 4, 6–8]. In addition, the panel reviewed two active registries and preclinical studies evaluating thermal injury depth, power-response dynamics, and procedural optimization [1, 2, 5, 11–13]. Unpublished data presented at international scientific and investigator meetings (not yet peer-reviewed) were also incorporated, including an aggregated report of 374 clinical cases [14, 15]. 

**Table 1:** Clinical Trials and Registries Evaluating GMA

Study	Treatment	Design	*N*	Grade III + SAE	NCT Number
Standalone fGMA					
Maselli et al. 2024 [3, 6]	fGMA	Single-center pilot	10	0 (0%)	NCT05578703
fGMA Registry ‡	fGMA	Multicenter prospective	35	0 (0%)	NCT06428617
Fundus + Corpus GMA					
COMET-2‡ [15]	GMA	Multicenter feasibility	10	2 (20%)	NCT05486338
GMA-AU‡ [15]	GMA	Pilot	12	0 (0%)	NCT05587491
GMA-BRASIL‡	GMA	Pilot	12	2 (16.7%)	NCT05574777
GMA for Weight Regain					
REVAMP‡ (interim)	GMA post-VSG	Prospective	8	0 (0%)	NCT06671119
MAINTAIN‡	GMA post–GLP-1	Prospective	0	N/A	NCT06734312
fGMA + ESG Combination					
Ablate Weight I (sequential) [4, 6]	fGMA → ESG	Prospective cohort	10	0 (0%)	NCT05578703
Ablate Weight II† [4]	fGMA + ESG	Prospective cohort	10	0 (0%)	NCT05992103
FULLNESS† [7] (interim)	ESG + fGMA	Prospective	22	0 (0%)	NCT06438510
fGMA + ESG Registry‡ (interim)	fGMA + ESG	Multicenter prospective	176	0 (0%)	NCT06420700
Stripe GMA + ESG (Durability)					
HAPCET-1† [8]	Stripe GMA + ESG vs. ESG	Single-center RCT	24	0 (0%)	NCT05559866
HAPCET-2†	Stripe GMA + ESG vs. ESG	Single-center RCT	45	0 (0%)	NCT05559866
TOTAL			**374**	**4 (1.1%)**	

† Peer-reviewed conference abstract. ‡ Manuscript under review, submitted, or unpublished registry data. Pooled safety estimates include non–peer-reviewed data and should be interpreted as exploratory.

## Drafting and Consensus Process

This framework was developed using a modified Delphi consensus process conducted between May 2025 and October 2025. Ten international experts with direct clinical or preclinical experience in GMA were invited to participate; 10 completed all three rounds (see Title Page for panel composition). Eligibility required authorship of a peer-reviewed GMA publication or active principal investigator status in a GMA clinical trial. An initial draft framework containing 8 safety statements organized across nine domains was developed by the coordinating authors (VK, CM) and circulated electronically. During each of three iterative rounds, panelists provided tracked edits, commentary, and anonymous votes on each statement. Agreement was defined as ≥ 75% of voting panelists endorsing a statement; recommendations were accepted if no substantive objections remained after the final review (Supplementary Figure S1) [16].

Given that the evidence base consists primarily of single-arm observational studies and pilot registries, formal GRADE assessment was not undertaken. Recommendation strength reflects collective expert agreement on procedural safety practices rather than formal certainty of evidence ratings. Recommendations were categorized by consensus strength rather than formal evidence grading: high consensus guidance (≥ 80% agreement) when panel agreement indicated the benefits clearly outweighed potential risks; moderate consensus guidance (60–79% agreement) when the benefit-risk balance was less certain or context-dependent; or low consensus guidance (51–59% agreement) when opinions were divided. This classification explicitly reflects the degree of panelist agreement and should not be equated with GRADE-based recommendation strength, which requires formal evidence appraisal.

### Conflict of Interest Management

Several panelists have received research funding, consulting fees, or equipment support from the manufacturer of the HybridAPC^®^ system. The following mitigation strategies were employed: (1) all financial relationships were disclosed to the panel prior to deliberations; (2) technical recommendations were derived from published literature and procedural experience rather than proprietary data; (3) voting on recommendations was conducted anonymously; (4) no Erbe employees participated in consensus meetings or voted on recommendations. The panel acknowledges that HybridAPC^®^ is the only commercially available integrated submucosal injection-APC system; alternative APC systems were not evaluated because they lack integrated high-pressure submucosal injection capability, the feature that enables simultaneous mucosal elevation and ablation within a single device. Standard (non-hybrid) APC systems require a separate injection catheter and device exchange, altering procedural workflow and potentially affecting the consistency of the submucosal cushion during ablation. Independent validation by centers without manufacturer relationships is warranted. The panel further recognizes that panelists who have invested in the development or early clinical application of GMA may be subject to confirmation bias, potentially favoring stronger endorsement of the technique. The anonymous voting structure and evidence-based deliberation process were specifically designed to attenuate this risk, though they cannot eliminate it entirely.

### Mechanistic Basis

The ghrelin-secreting X/A-like cells are concentrated in the oxyntic mucosa of the gastric fundus and proximal body, serving as the primary source of circulating ghrelin, a hormone with potent orexigenic effects that also stimulates gastric motility and modulates glucose metabolism [17–19]. 

Ghrelin accelerates gastric emptying, induces premature phase III‑like interdigestive contractions, and enhances cholinergic‑mediated vasovagal reflexes in the stomach. These prokinetic actions are mediated via ghrelin receptors on vagal afferents, enteric neurons, interstitial cells of Cajal, and smooth muscle, coordinating motility through both neural and muscle‑level pathways [20–24]. Ghrelin levels rise during fasting and fall after meals, playing a central role in meal initiation and energy homeostasis [25, 26]. 

By applying controlled thermal energy to this anatomically confined mucosal layer, GMA selectively devitalizes these endocrine cells while sparing deeper structures. This targeted mucosal ablation leads to a significant and sustained suppression of fasting ghrelin; typically 30–50% within weeks; as demonstrated in translational and early-phase clinical studies [3, 4, 12]. These hormonal effects are analogous to those observed after surgical sleeve gastrectomy (SG), where fundectomy eliminates this ghrelin-producing region.

In addition to endocrine modulation, GMA exerts a secondary mechanical effect through submucosal remodeling. Histological and imaging studies indicate that thermally induced fibrosis reduces fundic compliance, thereby limiting the stomach’s capacity to expand during meals. This reduction in distensibility enhances vagal afferent signaling, promoting early satiety and reduced meal size [3, 4]. Notably, these effects are achieved without anatomical resection or disruption, distinguishing GMA from both surgical and full-thickness endoscopic interventions.

Together, the hormonal and mechanical sequelae of GMA act synergistically to reduce appetite, prolong postprandial fullness, and lower caloric intake, supporting clinically meaningful weight loss. These physiological mechanisms correspond to observed improvements in weight loss, hunger scores, and satiety in early trials.

### Preclinical Development and Histologic Targets

The development of GMA was guided by translational models. In diet-induced obese rodents and randomized porcine trial, wide-field mucosal ablation of the gastric fundus resulted in significant reductions in body weight, visceral adiposity, hepatic steatosis, and circulating ghrelin levels [1, 2, 5]. 

Preclinical, ex-vivo, and cadaveric studies have consistently demonstrated that the combination of high-pressure submucosal saline injection and controlled energy delivery is critical to confining thermal injury during GMA. In the ex-vivo dose-finding study by Fayad et al., [5] thermal effects were restricted to the mucosa and superficial submucosa when ablation was performed over an elevated cushion, with no observed damage to the muscularis propria. Similarly, in the COMET Step 1 validation study (manuscript under review), histologic analysis in six patients who underwent ablation 3–5 days prior to sleeve gastrectomy confirmed complete mucosal devitalization in 100% of treated zones, with preservation of the muscularis propria in 97% [12]. These findings underscore that adequate mucosal elevation provides a protective thermal barrier, and that exceeding power thresholds or ablating unlifted or fibrotic tissue significantly increases the risk of deep tissue injury, including transmural necrosis.

**Fig. 1:**
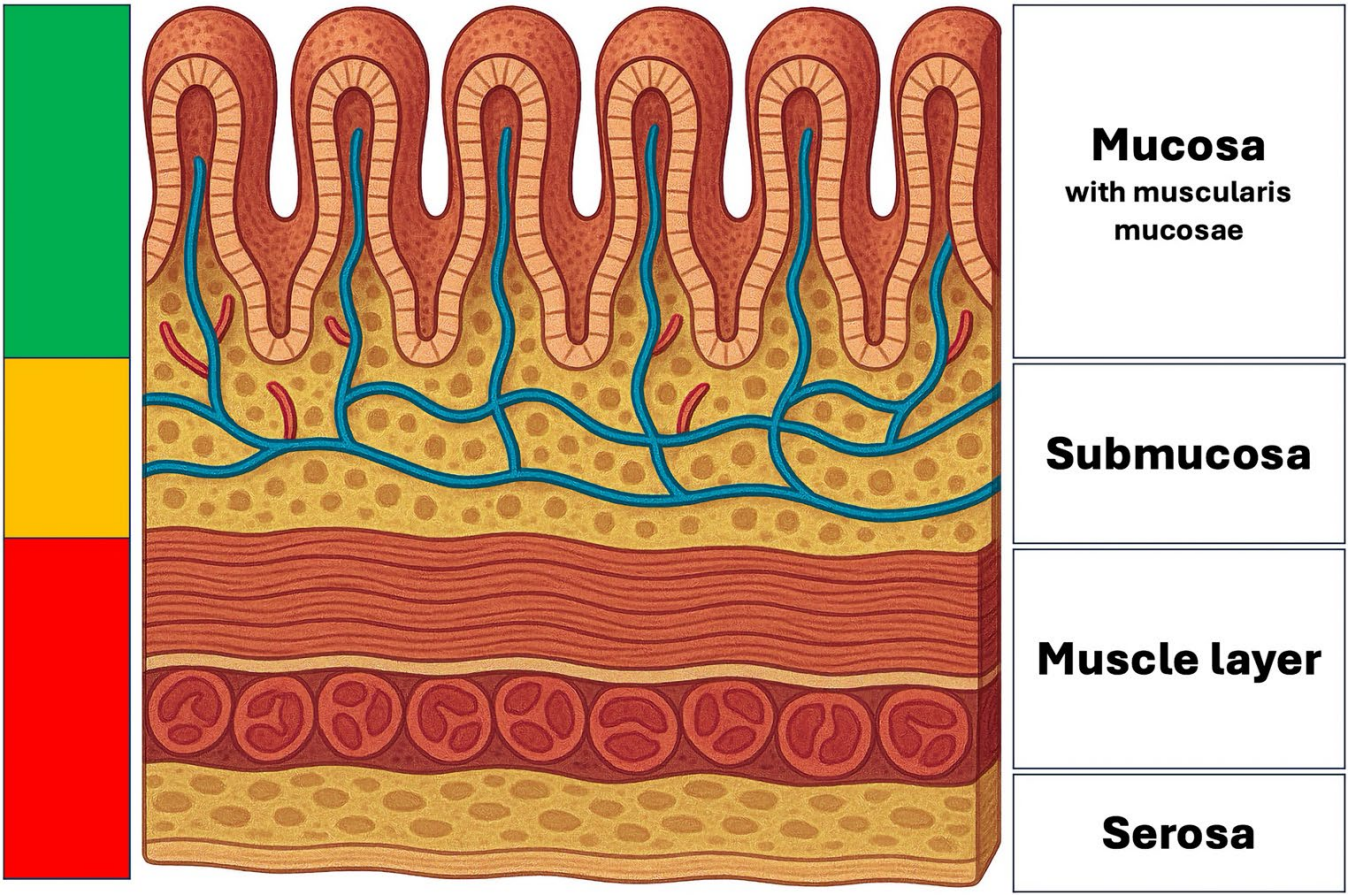
Layers of the stomach wall and thermal damage by GMA. The acceptance of the thermal damage by GMA is indicated by “traffic light” color bars. The goal is the ablation of the mucosa (green bar) which may also involve the upper two-thirds of the submucosa (yellow bar) which is considered as an additional safety space. Thermal damage that includes the last third of the submucosa and the muscle layer (red bar) is considered as too deep of an involvement of the stomach wall by GMA

To guide procedural safety, the COMET investigators proposed a three-tiered histologic classification, the “traffic light” model, in which the green zone represents intended mucosal and partial submucosal injury; the yellow zone represents submucosal injury without muscle involvement; and the red zone reflects transmural injury with muscularis damage, correlating with increased risk of perforation and adverse outcomes - Fig. 1.

This histologic framework has since been adopted to guide clinical dosing, endpoint recognition, and safety thresholds in ongoing studies and registries.

### Procedural Technique

Gastric mucosal ablation is an endoscopic procedure performed under general anesthesia with or without intubation using the HybridAPC^®^ or MOVIVA^®^ system, which combines high-pressure, needle-free submucosal injection with non-contact argon plasma coagulation (APC).

**Fig. 2:**
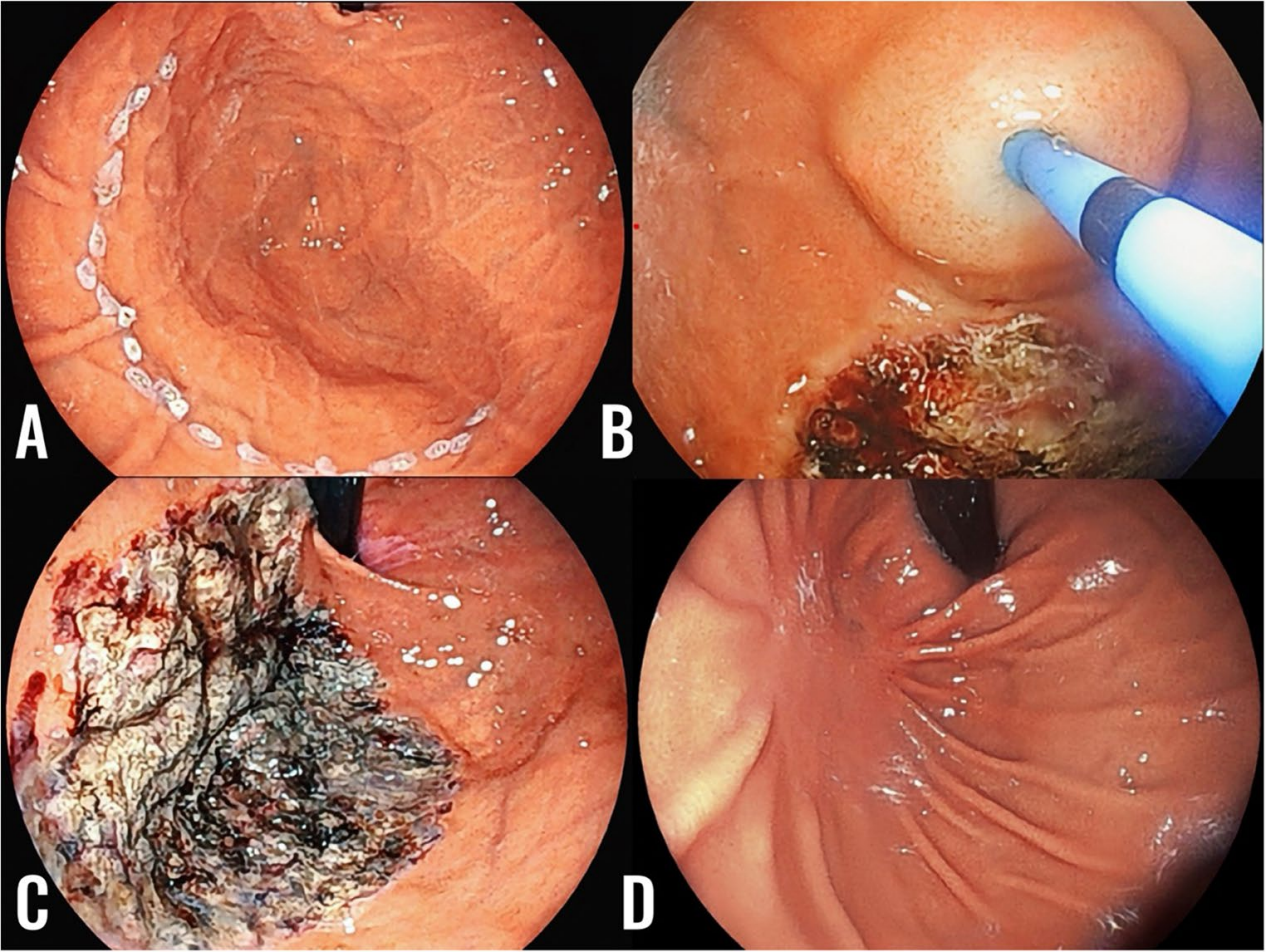
Sequential endoscopic views illustrating the four procedural phases of gastric mucosal ablation (GMA) of the fundus using only hybrid argon plasma coagulation (APC). (**A**) Mucosal boundary marking dots using APC to define the ablation area. (**B**) Submucosal injection followed by pulsed APC application (≤ 40 W) to lifted mucosa, producing targeted thermal devitalization; note the clear central elevation and preserved margin. (**C**) Completed ablation of the gastric fundus showing uniform devitalization and absence of deep charring or perforation. Darker spots are the result of superficial coagulated blood. (**D**) Endoscopic appearance of the treated area at 6-month follow-up demonstrating mucosal healing with characteristic fold convergence

Following endoscopic inspection, the target treatment area (typically the gastric fundus and/or greater curvature of the gastric body) is identified, and the border of the treatment zone is marked using brief APC pulses (20 W; Fig. 2A). Submucosal injection is then performed using a saline-dye solution (e.g., methylene blue or indigo carmine), typically requiring at least of 3 mL per injection site, as previously established as the minimum volume needed for sufficient cushion and sufficient thermal protection [13]. Injection pressure begins at Effect 20 (20 bar) and is increased stepwise, up to Effect 50, until visible mucosal lift is achieved (Fig. 2B). Total injectate volume per procedure ranges from 100 to 200 mL [4, 6]. 

**Fig. 3:**
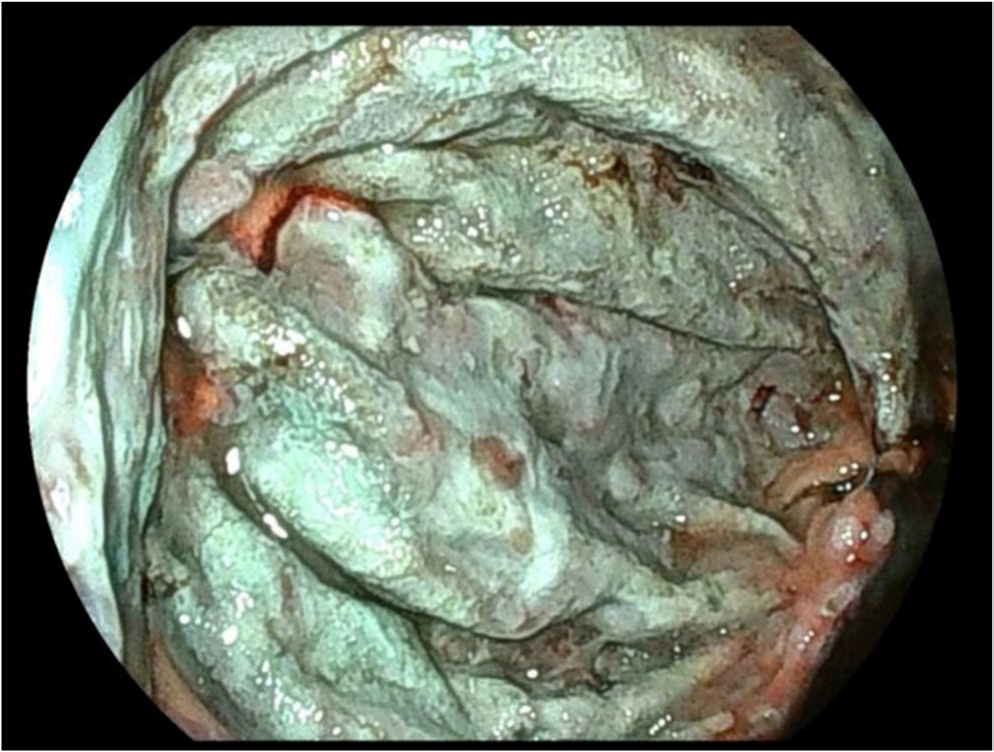
Endoscopic view of the gastric fundus near completion of GMA using pulsed APC ablation, demonstrating heterogeneous white-to-yellowish tissue discoloration

Once adequate elevation is confirmed, APC is applied in accordance with gastric location (typically lower wattage for thinner, proximal areas), using 30–40 W (pulsed APC, slow or fast) in steady, controlled motions until a uniform pale-yellow or ivory discoloration appears (Fig. 3), indicating effective thermal devitalization (Fig. 2B and C). Care should be taken to avoid direct contact between the APC probe and the mucosal surface and to avoid mucosal carbonization. The lift-and-ablate sequence is repeated across adjacent zones until the targeted mucosal surface is fully treated. In highly vascular regions, superficial capillary oozing may occur and coagulate under APC, resulting in a darker hue that should not be misinterpreted as deeper tissue injury (Fig. 2C). The HybridAPC probe allows seamless alternation between injection and ablation without device exchange. Treatment may be performed in a single session or staged, depending on operator preference and clinical context [3]. 

Argon flow is typically set at 0.8–1.0 L/min [4, 6, 15, 27]. Due to continuous gas insufflation during APC activation, frequent endoscopic aspiration or the use of an external suction system is required. Although not mandatory the use of a dual-channel endoscope may be preferred, as it allows for dedicated suction throughout the procedure. This approach minimizes the need to intermittently remove the APC probe for decompression, thereby improving procedural efficiency and workflow.

Abdominal wall tension should be frequently assessed via direct palpation, as rare cases of pneumoperitoneum have been reported, even in the absence of overt perforation [15, 27]. In one such case, pneumoperitoneum was attributed to gas retention secondary to delayed colonic transit and elevated intragastric pressure, which permitted gas to escape through the gastric wall [15, 27]. Pneumoperitoneum following APC has also been described in other anatomic regions. These cases are typically asymptomatic and can usually be managed conservatively [28–30]. 

### Clinical Application Scenarios

GMA has been explored to be performed as a standalone metabolic procedure or in combination with endoscopic sleeve gastroplasty (ESG). As a monotherapy, gastric fundal mucosal ablation (fGMA) produces a mean total-body weight loss of 7.7% at 6 months, accompanied by ~ 45% reductions in fasting plasma ghrelin, lower self-reported hunger scores, and a ~ 40% decrease in maximum tolerated meal volume [3]. 

GMA targeting both the fundus and the corpus has been and is being evaluated across multiple international pilot studies, including the COMET-2 (USA), GMA-BRASIL (Brazil), and GMA-AU (Australia) trials. In all three protocols were conservative in the total magnitude of surface area ablated (being pilot studies), overlapping stripes of mucosal ablation were applied to the fundus and proximal to mid-gastric body, aiming to treat up to 70% of the mucosal surface area. At 6-month follow-up, interim analyses demonstrated consistent TBWL across trials, with mean values of 9.6% (COMET-2), 8.5% (GMA-BRASIL), and 6.9% (GMA-AU), respectively. A pooled analysis yielded a combined estimate of 8.3% TBWL at 6 months (95% CI: 6.5–10.6%), with no significant heterogeneity observed (I² = 0%). Latest unpublished results on the first 8 patients from COMET-2 trial are showing signals that the effect persist up to 12 months with TBWL at 12.2% (95% CI: 4.8–19.6) [15]. Nevertheless, these are the first exploratory signals and should be considered with this in mind.

When combined with ESG, GMA appears to confer both hormonal and structural benefit. In this setting, ablation is typically applied to the fundus, while suturing targets the gastric body. The procedures may be performed either simultaneously or sequentially [4, 7]. 

In the ABLATE WEIGHT I and II studies, the simultaneous approach resulted in a TBWL of 23.5% at 12 months, while the sequential protocol reached 22.5% at 18 months [4]. Supporting these findings, the FULLNESS study, a prospective single-arm trial conducted at an Italian academic center in Europe, reported 18.6% TBWL at 12 months following simultaneous GMA and ESG after first 13 patients, with no serious adverse events and peak weight loss reaching 32% in some patients [7]. The ABLATE WEIGHT cohort also showed sustained reductions in hunger indices and standardized nutrient drink volumes, reinforcing the hypothesis that fundic mucosal devitalization enhances satiety signaling beyond mechanical restriction alone.

Beyond metabolic effects, GMA has been studied for improving ESG durability. In the randomized HAPCET-1 and − 2 trials, mucosal ablation along the suture line before ESG resulted in improved sleeve retention and reduced proximal dilation at 6 months, with more favorable sleeve geometry on endoscopy and MRI [8]. TBWL was similar between groups in both trials (HAPCET-1: 21.4% vs. 20.9%; HAPCET-2: 15.4% vs. 15.0% for GMA + ESG vs. GMA alone), likely because ablation did not involve the fundus. These data suggest that targeted mucosal ablation may enhance structural durability via fibrosis and tissue remodeling, effects that may require longer follow-up to translate into weight differences.

### Pathophysiology of Complications

GMA induces a controlled thermal injury to the mucosa layer, resulting in a devitalized epithelial layer that subsequently regenerates with altered hormonal and functional characteristics. The ablation process unfolds through a sequence of predictable histological stages: an initial phase of coagulative necrosis, followed by de-epithelialization with sloughing of necrotic mucosa, exposure of the submucosa, and ultimately re-epithelialization with submucosal remodeling. The depth and extent of thermal injury are determined by APC power settings, duration of application, and adequacy of submucosal lift.

The de-epithelialization phase, typically occurring between days 3 and 7 post-ablation, represents a window of heightened vulnerability. During this time, the exposed submucosa is susceptible to injury from the gastric environment, including hydrochloric acid and proteolytic enzymes. This “naked” surface remains unprotected until mucosal regeneration begins. The risk of injury is influenced by the depth of ablation, adequacy of acid suppression, and ongoing proteolytic secretion. If pharmacologic protection (e.g., PPI and sucralfate) is insufficient, or if solid foods are reintroduced prematurely, there is an increased risk of ulcer formation, delayed bleeding, and persistent inflammation.

Mechanical factors may also contribute to complications. For example, insufficient submucosal elevation or energy application over fibrotic or previously treated mucosa can result in unintended thermal extension into the muscularis propria or serosa, potentially leading to transmural necrosis, perforation, or perigastric fluid collections. Pre-procedurally, these risks highlight the importance of obtaining a detailed medical history, including prior gastric interventions such as explanted adjustable gastric bands or intragastric balloons, which can induce gastric and perigastric fibrosis and are not infrequent precursors to ESG in the chronic, progressive, and relapsing disease of obesity [31, 32]. Intra- and post-procedurally, these risks underscore the importance of precise procedural technique, judicious energy delivery, gradual dietary advancement, and robust pharmacologic support during the healing period.

A thorough understanding of these physiological and tissue-level dynamics is important for optimizing safety, minimize complications, and promote predictable mucosal regeneration following GMA.

### Safety Domains

Following detailed analysis of clinical and preclinical data and iterative consensus among experienced GMA investigators, the panel identified nine key domains forming the basis of a standardized risk mitigation bundle. These span procedural technique, post-procedural care, and operator training.

### Submucosal Injection

A high-pressure submucosal injection of cold saline (6–8 °C) should be performed before each ablation pass to create a well-defined fluid cushion. This cushion separates the mucosa from the muscularis propria and functions as a thermal barrier, limiting energy delivery to superficial layers. Ex vivo evidence indicate that a minimum volume of 3 mL per site reduces thermal injury to deeper layers [13]. The COMET Step 1 histopathology study [12] (manuscript under review) corroborated this finding in human tissue: muscularis propria was preserved in 97% (33/34) of ablation zones performed over an adequate submucosal cushion; the single zone with muscularis injury was attributed to insufficient submucosal injection. Furthermore, three of four serious adverse events in early clinical investigations occurred in regions with absent, inadequate, or unreplenished submucosal lift. While APC is routinely used in the gastrointestinal tract without submucosal elevation (e.g., for angiodysplasias or Barrett’s ablation), GMA differs fundamentally in that it targets wide-field, circumferential ablation of large mucosal surface areas, conditions that amplify cumulative thermal energy delivery and substantially increase the risk of deep tissue injury. Submucosal injection is therefore a mandatory procedural step.*(High consensus guidance; ≥80% agreement)*.

### APC Power Limitation

Argon plasma coagulation energy should generally be limited to 30–40 W using either standard or self-regulating modes. Although settings up to 50 W have been used safely in selected cases with an adequate submucosal cushion, dose-finding data from the COMET Step 1 study [12] (manuscript under review) demonstrate that 40 W and 50 W produce virtually identical ablation depths (22.0% ± 12.3% vs. 22.3% ± 6.5% of wall thickness, respectively), indicating that power escalation beyond 40 W confers no additional efficacy. The panel therefore recommends the 30–40 W range as the preferred operating window. Power settings for GMA should therefore be adjusted to gastric wall thickness by anatomic region: 30–35 W in the thin-walled fundus, 35–40 W in the proximal body, and up to 40 W in the distal body, where greater wall thickness allows higher energy delivery. *(High consensus guidance; ≥80% agreement)*

### Safety margin within the cushion

Ablation should be confined to the center of the lifted mucosal zone and should be discontinued at least 5 mm from the edge of the submucosal cushion. Ablating beyond this transition zone, where the submucosal cushion may be thinner or uneven, increases the likelihood of deep thermal injury, including delayed perforation. Maintaining a consistent margin within the cushion is important for procedural safety. *(High consensus guidance; ≥80% agreement)*

### Avoidance of Energy On Scarred or Un-lifted Mucosa

Areas of previously treated, fibrotic, or scarred mucosa may resist submucosal lifting and should not be subjected to ablation unless a new and adequate lift is achieved. Notably, one perforation was reported following application of 60 W APC to a fibrotic zone without reinjection [14]. Flat or non-elevating tissue should be avoided unless a prominent submucosal cushion is clearly established. *(Moderate consensus guidance; 60–79% agreement)*

### Frequent Argon Gas Evacuation

Argon is delivered at a rate of 0.8–1.0 L/min during procedures lasting 30–90 min, which can result in substantial intragastric gas accumulation. To prevent gastric distention, barotrauma, or gas extravasation, continuous and proactive gas management is important. Key mitigation strategies include frequent endoscopic suctioning, routine abdominal palpation to assess wall tension at regular intervals (e.g., every 5–10 min), and use of an endoscopic pressure regulator when available. These practices help maintain safe intraluminal pressures throughout the procedure. *(High consensus guidance; ≥80% agreement)*

### High-Dose Acid Suppression

Patients should be prescribed high-dose proton pump inhibitor therapy (e.g., omeprazole 40 mg twice daily) for at least four weeks and ideally up to twelve weeks following the procedure. Insufficient acid suppression has been associated with post-procedural complications including ulceration and bleeding in observational studies. Extended PPI therapy promotes mucosal healing and reduces the risk of delayed complications. Endoscopic evidence suggests that mucosal healing after wide-field GMA may require 8–12 weeks. *(Moderate consensus guidance; 60–79% agreement)*

### Sucralfate for Mucosal Protection

Sucralfate 1 g orally four times daily is recommended for four weeks following the procedure to enhance mucosal protection and promote healing. Although direct evidence in the context of GMA is limited, extrapolation from other mucosal injury settings supports its role in cytoprotection. Patients should be counseled on potential drug interactions and advised to separate administration from other oral medications. *(Moderate consensus guidance; 60–79% agreement)*

### Staged Dietary Advancement

Patients should follow a structured post-GMA diet beginning with 72 h of clear liquids, followed by gradual advancement through full liquids, purées, and soft solids. Solid foods should be delayed until at least Day 14, though longer delay may be warranted when GMA is combined with suturing procedures such as ESG. This approach minimizes risk of shear injury, acid stimulation, and mucosal disruption. *(Low consensus guidance; 51–59% agreement)*

### Operator Credentialing

Credentialing for GMA should prioritize demonstrated proficiency in key technical skills, particularly submucosal injection and precise, controlled application of thermal energy. Inexperienced operators should complete ex-vivo model training and a minimum of 3–5 supervised clinical cases, with stepwise adherence to safety protocols. Objective competency benchmarks, such as independent achievement of adequate submucosal lift, appropriate tissue color endpoints, and correct identification of when to discontinue ablation, should be documented before unsupervised practice. The optimal case volume for credentialing remains to be established through prospective assessment. The suggested 3–5 proctored case volume is illustrative; institutions should prioritize competency-based assessment, such as consistent achievement of adequate submucosal lift and correct recognition of tissue endpoints, over a fixed numerical threshold alone. *(Moderate consensus guidance; 60–79% agreement)*

### Periprocedural Care

Periprocedural care should follow standard advanced endoscopy protocols: short-term antiemetics and analgesics as needed per locally established practice; strict avoidance of NSAIDs for 12 weeks; no routine antibiotics or systemic steroids; and routine monitoring that recognizes modest tachycardia or low-grade fever as physiological during the first 72 h. Intramural or limited extramural gas on post-procedure imaging is usually benign and warrants intervention only if clinical signs of perforation emerge. GMA should be deferred in patients who cannot safely discontinue anticoagulation or antiplatelet therapy for the full 12-week healing period.

Together, these nine domains define the core safety framework for GMA and are summarized in Table 2. The panel recommends that these measures be incorporated into all future investigational protocols and registries.

**Table 2:** Consensus Recommendations for Risk Mitigation in Gastric Mucosal Ablation

Domain	Recommendation	Guidance Tier	Consensus Strength	Rationale
Submucosal injection	**We recommend** performing cold (6–8 °C) saline submucosal injection before each ablation pass, with at least 3 ml per injection site.	High consensus	High consensus (≥ 80%)	Ensures mucosal elevation and thermal insulation; enhances submucosal cushion thermal protection
Energy setting	**We recommend** APC limited to ≤ 40 W	High consensus	High consensus (≥ 80%)	Prevents deep thermal injury; thresholds validated in preclinical models
Treatment safety margin	**We recommend** maintaining an untreated margin of at least 5 mm from the edge of the cushion	High consensus	High consensus (≥ 80%)	Prevents ablation of transitional zones lacking submucosal protection
Mucosal condition	**We recommend** avoiding energy delivery over scar tissue or un-lifted mucosa	Moderate consensus	Moderate consensus (60–79%)	Scarred or flat areas are associated with unpredictable thermal spread
Argon evacuation	**We recommend** frequent argon evacuation with simultaneous monitoring of abdominal distention	High consensus	High consensus (≥ 80%)	Prevents over-distention of the gastric lumen, argon extravasation, and barotrauma
Acid suppression	**We suggest** administering high-dose PPI (e.g. 40 mg BID) for 8–12 weeks	Moderate consensus	Moderate consensus (60–79%)	Reduces ulcer formation and delayed bleeding; supported by observational data
Mucosal protection	**We suggest** prescribing sucralfate 1 g QID for 4 weeks	Moderate consensus	Moderate consensus (60–79%)	Provides topical barrier during healing; extrapolated from other APC applications
Diet advancement	**We suggest** implementing a staged reintroduction of food over ≥ 14 days (72 h clear liquids → full liquids → purée → soft solids)	Low consensus	Low consensus (51–59%)	Minimizes chemical and mechanical stress on ablated mucosa
Operator training	**We recommend** initial proctoring to achieve procedural proficiency	Moderate consensus	Moderate consensus (60–79%)	Learning curve analysis shows improved adherence and reduced AE rate after 3 to 5 cases

### Safety Outcomes

The safety profile of GMA using hAPC has been evaluated across multiple early-phase clinical trials and registries. As of February 2026, a total of 374 procedures have been performed, with safety data pooled from all trials and registries in which GMA was performed either as a standalone intervention or in combination with endoscopic sleeve gastroplasty (ESG). The most frequently reported adverse events were mild and self-limited.

Across COMET-2, GMA-BRASIL, and GMA-AU, a total of 424 adverse events were recorded among 34 patients (374 total procedures across all studies); individual patients could experience multiple events, and 94–99% of events were classified as Clavien-Dindo Grade I. Most common: abdominal pain (18–30%), nausea (12–20%), bloating/cramping (4–12%). Events were self-limited within 48–72 h. Importantly, in combined procedures with ESG, clinical experience suggests that ESG and not GMA is the primary driver of AEs. Finally, patients undergoing GMA should be made aware of blue coloration of urine in the 48 h after the procedure from expected excretion of the blue dye components of the saline lift solution absorbed during the procedure.

Four serious adverse events (SAEs; Clavien-Dindo Grade III+) occurred among 374 procedures (1.1%) [14, 15]. All four events occurred in protocols involving ablation of both the gastric fundus and body during the early adoption phase. No SAEs have been documented in fundus-only or fundus GMA + ESG protocols to date. The SAEs comprised: (1) bleeding ulcer requiring endoscopic intervention (*n* = 2); (2) perforation with perigastric fluid collection requiring drainage (*n* = 1); (3) pneumoperitoneum without macroscopic perforation, managed by diagnostic laparoscopy (*n* = 1). Contributing factors in all cases included protocol deviations: insufficient treatment margin (*n* = 1), energy application exceeding 40 W (*n* = 1), insufficient gas management (*n* = 1) or combination of insufficient submucosal injection and excessive energy application (*n* = 1). Serious adverse events were classified according to the Clavien-Dindo grading system and independently adjudicated by a Data Safety Monitoring Board (DSMB) across all studies reporting SAE outcomes.

Following implementation of the standardized risk mitigation bundle, across five centers between May 2024 and the end of the observation period, 120 consecutive procedures were completed with documented protocol adherence and without Grade III or higher adverse events. In addition, early experience in the European Union, encompassing approximately 120 further procedures, reported no serious adverse events. Although uncontrolled, this pooled observational experience supports the potential effectiveness of the proposed safety measures.

## Discussion

Gastric mucosal ablation using HybridAPC^®^ is an investigational technique that modulates appetite-regulating endocrine pathways by selectively devitalizing ghrelin-producing mucosa. Early clinical trials have demonstrated technical feasibility and metabolic effects that may approach those of surgical sleeve gastrectomy when combined with endoscopic sleeve gastroplasty.

GMA carries procedural risks when key safety parameters are not followed. The observed SAE rate of 1.1% occurred predominantly during early operator experience and was associated with identifiable technical deviations. This rate should be interpreted in context: published meta-analyses report SAE rates of 1.0–2.5% for ESG, 0.3–1.9% for intragastric balloons, and 1.0–5.4% for laparoscopic sleeve gastrectomy in comparable populations [31–34]. Direct comparison is limited by heterogeneity in study designs, patient selection, and outcome definitions; the absence of prospective head-to-head trials represents a critical evidence gap.

To mitigate procedural risks, this multidisciplinary panel developed a standardized framework organized around nine interrelated safety domains: cold submucosal injection, APC power limitation (≤ 40 W), energy confinement within the lifted cushion, avoidance of ablation over fibrotic or un-lifted tissue, frequent argon gas evacuation, pharmacologic support (PPI and sucralfate), staged dietary advancement, and operator credentialing.

There are several limitations that need to be acknowledged. The evidence base consists of small, single-arm observational studies and pilot registries with follow-up limited to 6–18 months; no randomized controlled trials comparing GMA with sham, pharmacotherapy, or established interventions have been completed. Several references represent manuscripts under review or conference presentations and have not yet undergone independent peer review [12, 14, 15, 27]; conclusions drawn from these data should be considered preliminary. Consensus strength therefore reflects expert agreement rather than formal GRADE-based evidence appraisal. Safety data were pooled across heterogeneous protocols differing in treatment extent, energy settings, and operator experience, limiting procedure-specific risk attribution. Panel members included investigators with direct procedural experience and, in some cases, financial relationships with the device manufacturer. Although mitigation strategies were employed, residual bias, including publication bias, selection bias, and confirmation bias among investigators personally invested in the technique, cannot be entirely excluded. Data originate largely from high-volume academic centers, limiting generalizability to community practice and less experienced operators. Recommendations apply specifically to the HybridAPC^®^ system and should not be extrapolated to other energy platforms without independent validation. Long-term safety beyond 18 months remains uncertain, underscoring the need for continued registry surveillance and prospective follow-up studies.

In conclusion, this consensus-based framework provides provisional safety guidance for clinical investigation of GMA. This guidance establishes preliminary safety standards and may serve as a model for the systematic introduction of the emerging endoscopic metabolic therapies. The panel recommends that investigators apply the complete risk mitigation framework within clinical trials, registries, and structured investigational programs.

## Supplementary Information

Below is the link to the electronic supplementary material.

Figure S1

High Resolution Image (TIF 1.21 MB)

## Data Availability

No datasets were generated or analysed during the current study.
