# Supplementary figures and images for: Safety Framework for Gastric Mucosal Ablation (GMA) Using Hybrid Argon Plasma Coagulation (hAPC): International Expert Consensus
Source: Obes Surg. 2026 May 16;36(7):3586–97. doi: 10.1007/s11695-026-08685-3 (PMC13323200; doi:10.1007/s11695-026-08685-3)

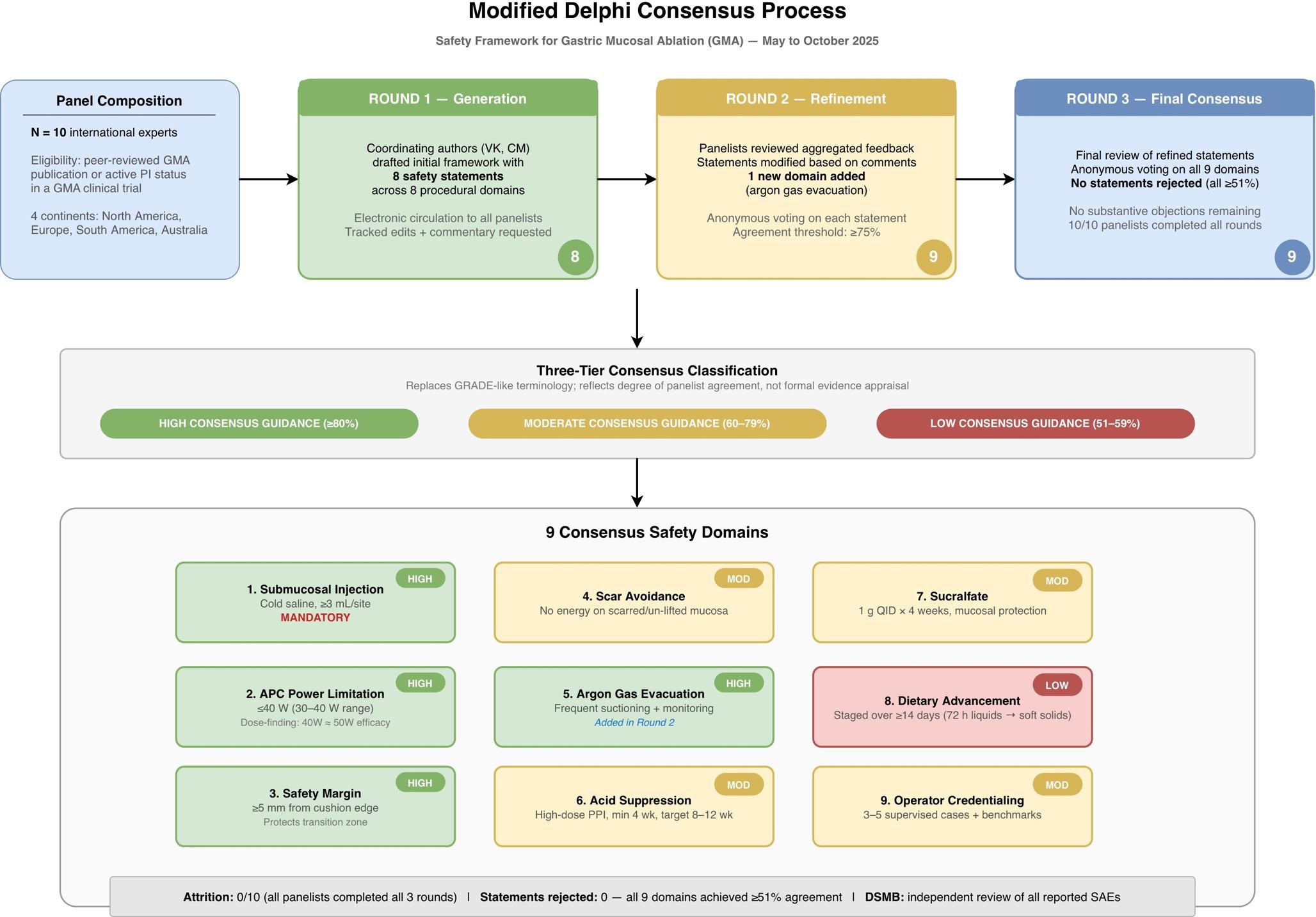

Supplement: Supplementary file 1 — Figure S1 [file 11695_2026_8685_Fig4_ESM.jpg]

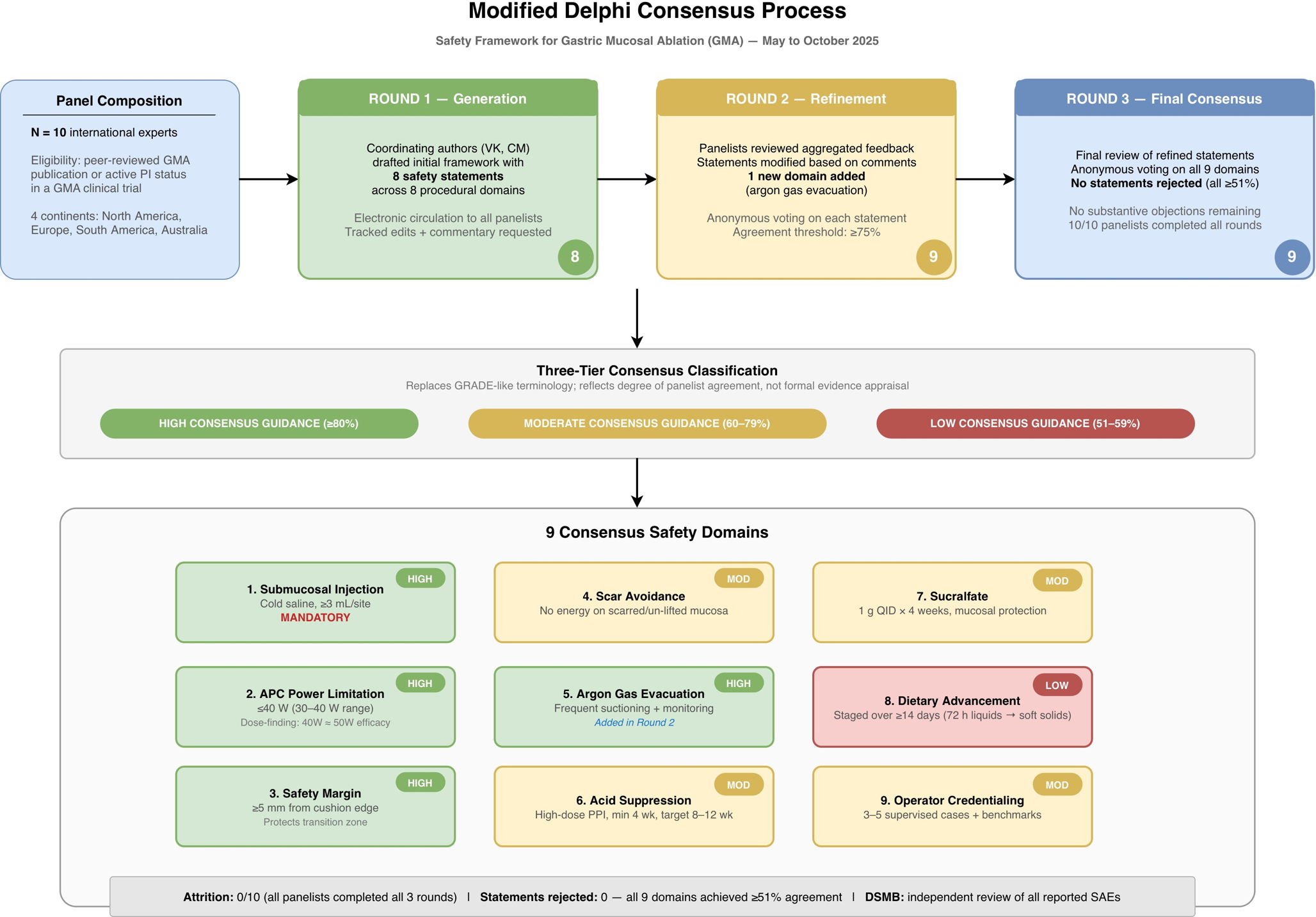

Supplement: Supplementary file 2 — High Resolution Image (TIF 1.21 MB) [file 11695_2026_8685_MOESM1_ESM.tif]
